# Molecular characterization of porcine reproductive and respiratory syndrome virus identified in 2021 from Nepal

**DOI:** 10.3389/fvets.2024.1267571

**Published:** 2024-04-02

**Authors:** Meera Prajapati, Manita Aryal, Yanmin Li, Zhidong Zhang, Madhav Prasad Acharya, Stephanie Clive, Jean-Pierre Frossard

**Affiliations:** ^1^National Animal Health Research Centre, Nepal Agricultural Research Council, Lalitpur, Nepal; ^2^Central Department of Microbiology, Tribhuvan University, Kathmandu, Nepal; ^3^College of Animal Husbandry and Veterinary Medicine, Southwest Minzu University, Chengdu, China; ^4^Animal and Plant Health Agency, Addlestone, United Kingdom

**Keywords:** ORF5, ORF7, Nsp2, PRRS, PRRSV

## Abstract

Porcine reproductive and respiratory syndrome (PRRS), an important viral disease of swine caused by PRRS virus (PRRSV) was first confirmed in Nepal in 2013. Since then, the virus has spread throughout the country and has now become endemic affecting the pig production nationally. However, molecular characterization of circulating strains has not been done in Nepal yet. In the present study, serum samples were collected from outbreak areas of different districts of Nepal and samples positive for PRRSV by ELISA were sent to Animal and Plant Health Agency (APHA), United Kingdom for sequence analysis. Out of 35 samples that were sent to APHA, only one sample was found positive by PCR and subjected to sequence analysis based on ORF5, ORF7 and Nsp2. The results from the phylogenetic analysis demonstrated that the PRRSV strain belongs to PRRSV-2 and lineage 8 strain. The sequences from the Nepalese PRRSV strain revealed a high degree of similarity with the strains isolated from India, China and Vietnam, with the closest genetic relatedness to the Indian isolates from 2020 and 2018. This is the first study on molecular characterization of PRRS virus circulating in Nepal. Further studies on strains circulating in Nepal are very essential to understand the virus diversity, its spread and evolution.

## 1 Introduction

Porcine reproductive and respiratory syndrome (PRRS) is an important infectious viral disease of swine which has caused huge economic impact in pig production worldwide. The disease is caused by PRRS virus (PRRSV) which is an enveloped, positive sense single-stranded RNA virus within the family *Arteriviridae* (1). PRRSV genome consists of nine open reading frames (ORFs) and is approximately 15 kb long (2). The nonstructural proteins are encoded by ORF 1a and 1b which are related to replication whereas ORF 2–7 encode structural proteins GP2, GP2b, E, GP3, GP4, GP5, GP5a, M, and N, respectively (3). Nsp2 is the largest PRRSV nonstructural proteins and recognized as a variable gene where frequent mutations, insertions, or deletions are observed. This distinctive feature makes Nsp2 a valuable marker for tracking the genetic changes and evolution of PRRSVs (4). ORF5 encodes the major envelope glycoprotein GP5, which is involved in viral attachment to cells and contains a neutralization epitope. ORF5 exhibits marked genetic variations compared to other genes and is therefore widely used for phylogenetic analyses (5, 6). Nucleocapsid protein (N) encapsidating the viral RNA genome is highly immunogenic in infected animals and is encoded by the ORF7 gene (7).

In the mid to late 1980s, PRRSV was reported for the first time in North America followed by Europe in 1990 (8). The two species of PRRSV, i.e., PRRSV-1 (previously European genotype) and PRRSV-2 (previously North American genotype) cause similar clinical infection to pigs though they have approximately 60% nucleotide identity at the genome level and 50–80% amino acid similarity (9, 10). In addition, within species, the Nsp2 and ORF5 genes vary considerably with sequence differences as high as 20% (10–12). Therefore, ORF5 and Nsp2 are preferred for studying the evolution and molecular epidemiology research on PRRSV (13–15). Based on molecular characterization of ORF5, PRRSV-2 have been further categorized into 9 lineages with several sublineages of each lineage and PRRSV-1 into three subtypes (subtype 1–3) (16).

PRRSV is transmitted through direct contact between infected pigs via ingestion of contaminated feed, inhalation of infected aerosols by coitus and semen of infected pigs (17). Indirect transmission occurs through fomite (18). Clinically the disease is manifested in two forms; reproductive and respiratory form (19). The reproductive signs include birth of still born piglets, abortions, mummified fetuses and weak born pigs. Respiratory signs include pneumonia, reduced feed intake, debilitation, chronic recurring illness and often high mortality.

Clinical cases of PRRS was confirmed for the first time in 2013 when the pig industry was booming in Nepal (20). In the same year, India also reported its first outbreak of PRRSV in pig population of Mizoram state (21). Since then, the virus has spread throughout the country affecting the pig production nationally. However, not much molecular epidemiological studies have been carried out. In addition, there is no active surveillance of PRRS to generate information on detection and distribution of disease or infection in the animal population. This study aimed to investigate the presence of PRRSV and characterize based on ORF5, ORF7 and Nsp2 sequence analysis.

## 2 Methodology

### 2.1 Sampling

**Figure 1:**
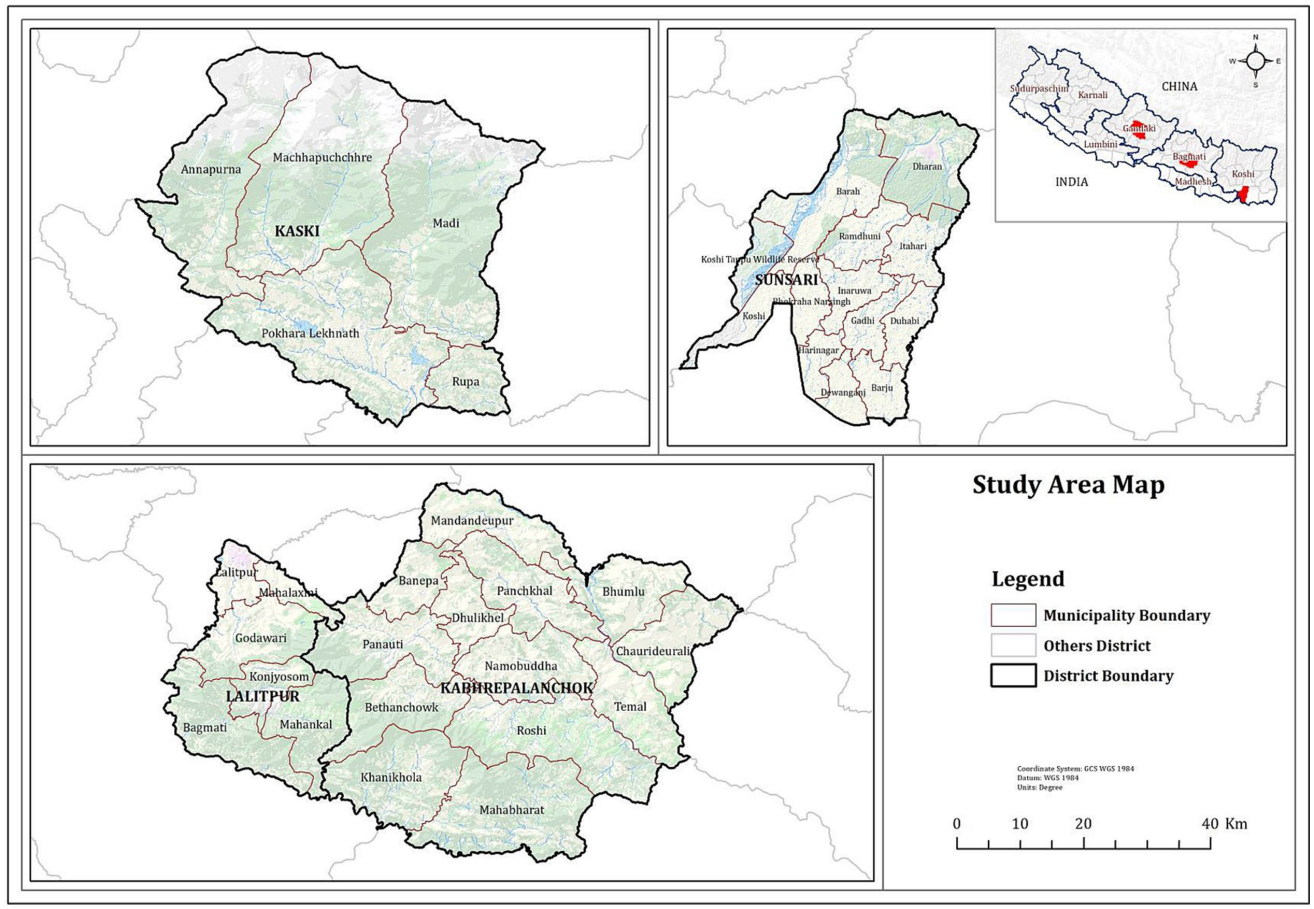
Map of Nepal showing sample collected districts.

Altogether 180 serum samples were collected from pig farms in four districts, namely Kaski, Lalitpur, Kabhrepalanchok and Sunsari of Nepal (see Figure 1) which had a history of PRRSV outbreaks at different time periods. The farmers’ consent was taken before collecting samples. Animals were controlled and the blood was withdrawn in a humane way using a sterile 3 mL syringe. Samples were collected from the age group of 4 months to 2 years. Serum samples were tested to detect PRRSV antibodies using a commercial ELISA kit (22). Thus, collected serum samples were stored for further studies and only the 35 sera samples which were detected positive by ELISA was sent to the Animal and Plant Health Agency, UK for genome sequence analysis.

### 2.2 RNA extraction, PCR amplification and sequencing

Nucleic acids from all samples were extracted using the KingFisher™ Flex Purification System (Thermo Fisher Scientific) and the MagMAX^™^ CORE Nucleic Acid Purification Kit (Applied Biosystems) following the manufacturer’s instructions for low cell content samples using the protocol MagMAX_CORE_Flex_no_heat.bdz.

All samples were tested for the presence of PRRSV RNA by performing a RT-qPCR using the VetMAX^™^ PRRSV EU & NA 2.0 Kit (Life Technologies) following the manufacturer’s instructions on an Agilent Aria machine.

For positive samples sequencing PCRs were carried out for ORF5, ORF7 and Nsp2. In brief, a conventional PCR was performed as a single step RT-PCR reaction using the Invitrogen^™^ Superscript^™^ IV One-step RT-PCR kit, with cycling conditions: 50°C for 10 min, 98°C for 2 min, (98°C for 10 s, 61°C for 10 s, 72°C for 30 s) × 40, 72°C for 5 min. The products were visualized on a 1.8% agarose gel using SYBR Safe gel stain (Thermo Fisher Scientific). The primers for ORF5, ORF7 and Nsp2 were listed in Table 1. Sanger sequencing was carried out using the same primers as for amplification, with cycle sequencing using the Big Dye Terminator v3.1 Cycle Sequencing Kit, followed by sequencing by capillary electrophoresis on an AB 3130xl instrument. The sequences generated during this study have been deposited in NCBI (OP037986.1, OP037987.1, and PP227416).

**Table 1:** Primers used for porcine reproductive and respiratory syndrome virus (PRRSV) detection.

Primer	Sequence (5′-3′)	Product size (bp)
ORF5-F	TGGCAATTTGAATGTTCAAGTATG	602
ORF5-R	CTGTGCTATCATTGCAGAAGTCGT	
ORF7-F	GCTGTTAAACAGGGAGTGG	372
ORF7-R	CGCCCTAATTGAATAGGTGAC	
Nsp2-F1	AAGTTAATGGTCTTCGAGCAGTG	1,544
Nsp2-R1	CTTTGTTCTTCGAGGTTGAACTCT	
Nsp2-F2	AACACCCAGGCGACTTCAGA	1,789
Nsp2-R2	TCTCATTAGGAGCAGTTCTTACACA	
Nsp2-F3	ATCATCGACTCTGGCGGGC	676
Nsp2-R3	ACCCGGAGAATAACCACTGT	

### 2.3 Phylogenetic and sequence analysis of PRRSV strains

**Figure 2:**
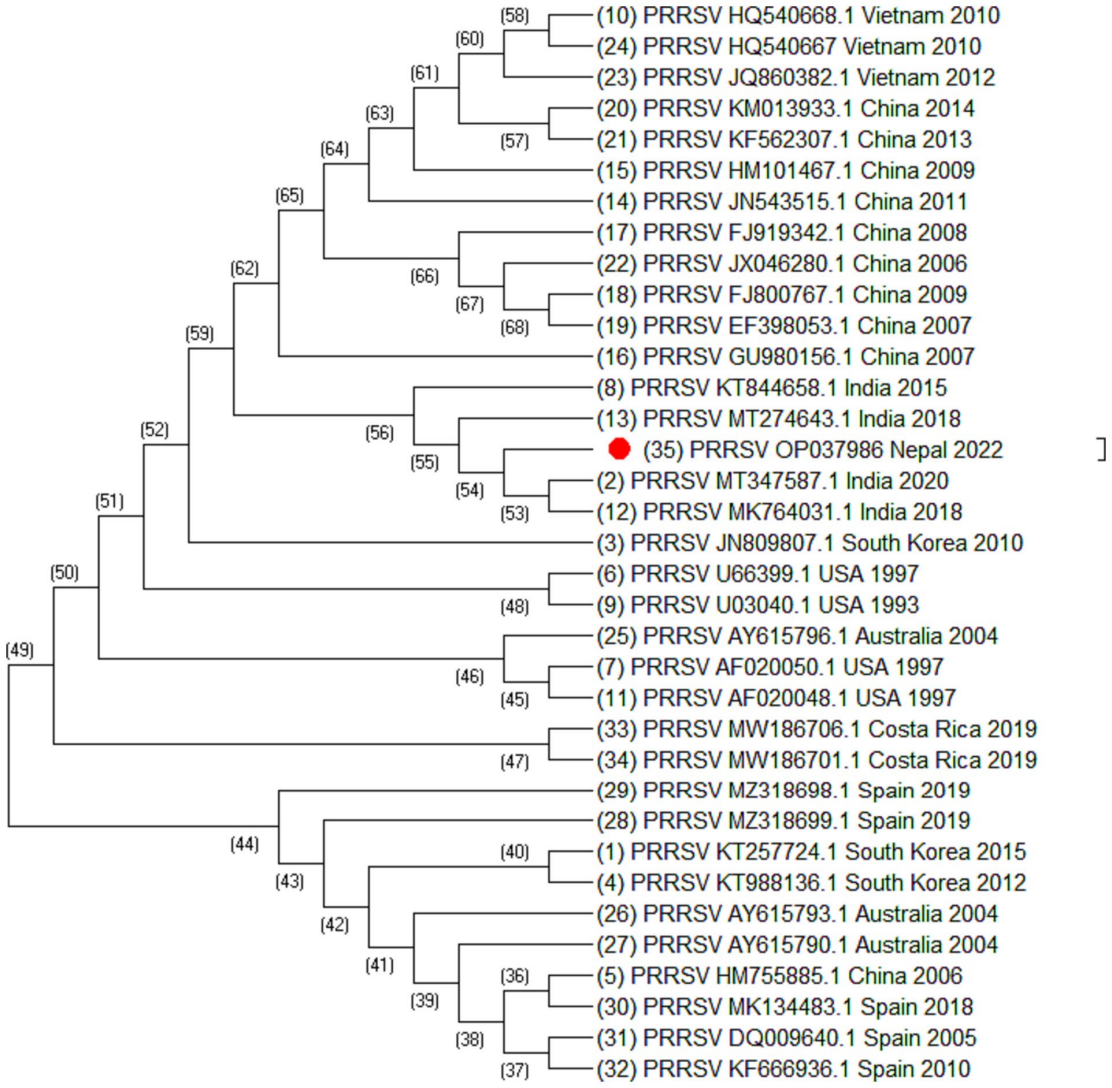
Phylogenetic tree of PRRSV based on ORF5 sequences. Altogether 35 sequences were used for analysis. The labels represent the name of virus followed by NCBI number country of isolation and year of isolation. The strain from this study is highlighted in red.

**Figure 3:**
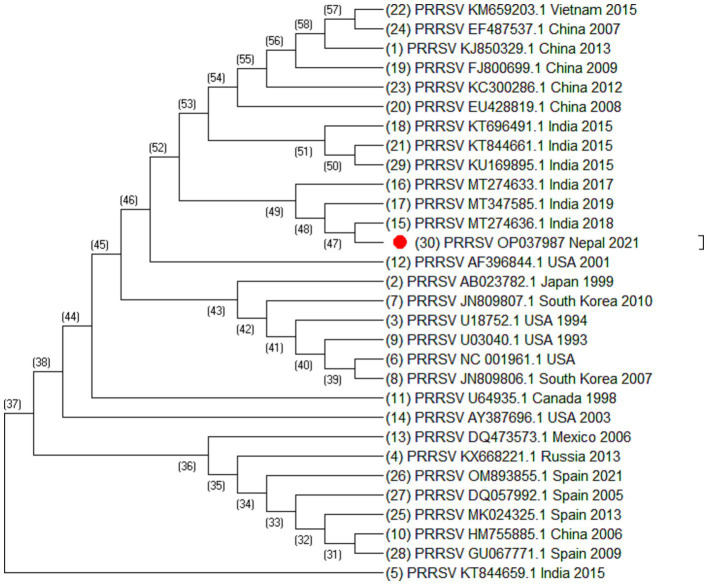
Phylogenetic tree of PRRSV based on ORF7 sequences. Altogether 30 sequences were used for analysis. The labels represent the name of virus followed by NCBI number country of isolation and year of isolation. The strain from this study is highlighted in red.

**Figure 4:**
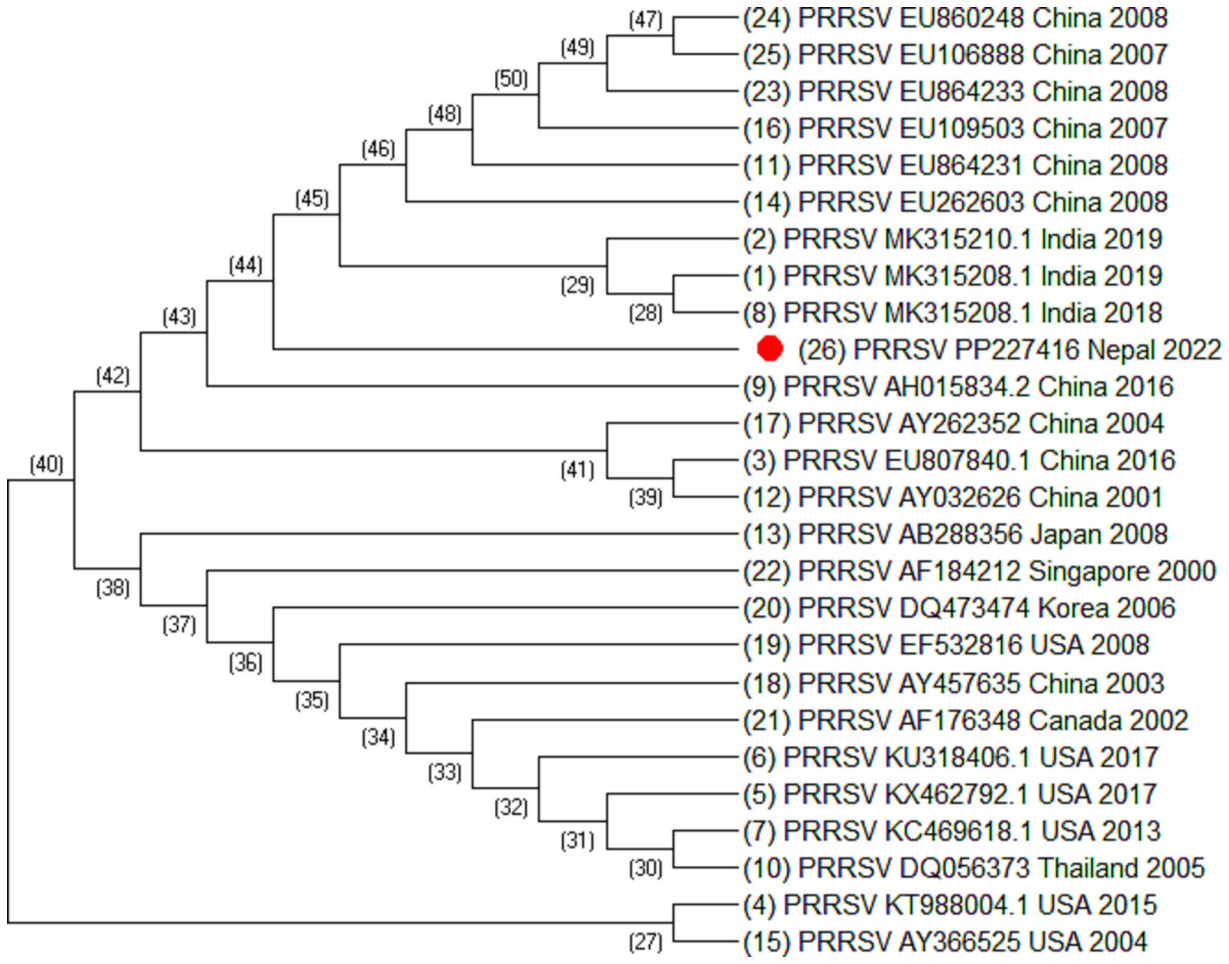
Phylogenetic tree of PRRSV based on Nsp2 sequences. Altogether 25 sequences were used for analysis. The labels represent the name of virus followed by NCBI number country of isolation and year of isolation. The strain from this study is highlighted in red.

Altogether, 35 sequences of ORF5 and 30 sequences of ORF7 of PRRSV and 25 sequences of Nsp2 were analyzed. The sequences include the sequence generated during this study together with sequences downloaded from NCBI. The sequences used in this study are listed in Tables 2–4. Phylogenetic and molecular analysis were carried out using MegaX software (23) and the phylogenetic trees were generated (see Figures 2–4) (neighbor joining method with 1,000 bootstrap replicates).

**Table 2:** List of PRRSV ORF5 sequences.

S. No.	Gene accession number	Countries	Year of isolation	Date of sequence deposition in NCBI	Place of isolation
1	KT257724.1	South Korea	2015	06-JUL-2015	South Korea
2	MT347587.1	India	2019	2020	Assam
3	KT988136.1	South Korea	2012	31-JAN-2016	South Korea
4	JN809807.1	South Korea	2010	05-NOV-2012	South Korea
5	HM755885.1	China	25-AUG-2006	22-NOV-2010	China
6	U66399.1	USA	1997	02-JUL-1997	USA
7	AF020050.1	USA	1997	20-APR-1998	North America
8	KT844658.1	India	02-JUN-2015	12-MAR-2016	India
9	U03040.1	USA	29-OCT-1993	24-MAY-1995	USA
10	HQ540668.1	Vietnam	03-NOV-2010	17-NOV-2010	Vietnam
11	AF020048.1	USA	19-AUG-1997	20-APR-1998	USA
12	MK764031.1	India	2018	30-OCT-2019	Assam
13	MT274643.1	India	2018	06-APR-2021	Kerala
14	JN543515.1	China	03-AUG-2011	28-DEC-2011	Hangzhou
15	HM101467.1	China	AUG-2009	15-JUN-2010	Sichuan
16	GU980156.1	China	JUN-2007	25-APR-2010	Guangzhou
17	FJ919342.1	China	OCT-2008	25-MAY-2009	Chongqing
18	FJ800767.1	China	03-MAR-2009	10-SEP-2009	Beijing
19	EF398053.1	China	28-JAN-2007	28-FEB-2007	Henan
20	KM013933.1	China	14-JUN-2014	04-OCT-2014	Wuzhou
21	KF562307.1	China	18-AUG-2013	10-JUL-2014	Nanjing
22	JX046280.1	China	2006	31-JUL-2012	Guangxi
23	JQ860382.1	Vietnam	2012	19-JUN-2012	Vietnam
24	HQ540667	Vietnam	3-NOV-2010	17-NOV-2010	Vietnam
25	AY615796.1	Australia	03-MAY-2004	09-JUN-2005	Australia
26	AY615793.1	Australia	3-MAY-2004	3-MAY-2004	Australia
27	AY615790.1	Australia	03-MAY-2004	09-JUN-2005	Australia
28	MZ318699.1	Spain	2019	05-APR-2022	Spain
29	MZ318698.1	Spain	2019	05-APR-2022	Spain
30	MK134483.1	Spain	6-NOV-2018	13-FEB-2019	Spain
31	DQ009640.1	Spain	15-APR-2005	19-JAN-2006	Spain
32	KF666936.1	Spain	22-MAR-2010	11-MAR-2014	Spain
33	MW186706.1	Costa Rica	2019	07-JUL-2021	Costa Rica
34	MW186701.1	Costa Rica	2019	07-JUL-2021	Costa Rica
35	OP037986.1	Nepal	2022	26-DEC-2022	Nepal

The sequences are arranged according to the gene accession number, countries, year of isolation, year of sequence deposition, and place of isolation.

**Table 3:** List of PRRSV ORF7 sequences.

S. No.	Gene accession number	Countries	Year of isolation	Date of sequence deposition in NCBI	Place of isolation
1	KJ850329.1	China	25-MAY-2013	25-MAY-2013	Guangxi
2	AB023782.1	Japan	16-FEB-1999	24-FEB-1999	Japan
3	U18752.1	USA	15-DEC-1994	27-JAN-1996	USA
4	KX668221.1	Russia	2013	08-MAY-2019	Russia
5	KT844659.1	India	10-JUN-2015	12-MAR-2016	Mizoram
6	NC_001961.1	USA	04-FEB-1998	13-AUG-2018	USA
7	JN809807.1	South Korea	2010	05-NOV-2012	South Korea
8	JN809806.1	South Korea	2007	05-NOV-2012	South Korea
9	U03040.1	USA	29-OCT-1993	24-MAY-1995	USA
10	HM755885.1	China	25-AUG-2006	22-NOV-2010	China
11	U64935.1	Canada	01-MAY-1998	04-AUG-1998	Canada
12	AF396844.1	USA	03-JUL-2001	01-AUG-2004	USA
13	DQ473573.1	Mexico	04-APR-2006	07-MAY-2007	Mexico
14	AY387696.1	USA	11-SEP-2003	01-MAY-2004	USA
15	MT274636.1	India	2018	06-APR-2021	Kerala
16	MT274633.1	India	2017	06-APR-2021	Kerala
17	MT347585.1	India	18-AUG-2019	23-AUG-2020	Assam
18	KT696491.1	India	02-JUN-2015	17-FEB-2016	Mizoram
19	FJ800699.1	China	03-MAR-2009	10-SEP-2009	Beijing
20	EU428819.1	China	25-JAN-2008	01-MAR-2008	Guangxi
21	KT844661.1	India	09-JUN-2015	12-MAR-2016	Mizoram
22	KM659203.1	Vietnam	26-SEP-2014	19-JAN-2015	Vietnam
23	KC300286.1	China	2012	20-MAR-2013	China
24	EF487537.1	China	9-MAR-2007	21-APR-2007	Shandong
25	MK024325.1	Spain	2013	01-APR-2021	Spain
26	OM893855.1	Spain	JUL-2021	05-APR-2022	Spain
27	DQ057992.1	Spain	11-MAY-2005	26-JUL-2016	Spain
28	GU067771.1	Spain	07-OCT-2009	24-JUL-2016	Spain
29	KU169895.1	India	11-JUN-2015	03-MAY-2016	India
30	OP037987	Nepal	26-DEC-2022	26-DEC-2022	Nepal

The sequences are arranged according to the gene accession number, countries, year of isolation, year of sequence deposition and place of isolation.

**Table 4:** List of PRRSV Nsp2 sequences.

S. No.	Gene accession number	Countries	Year of isolation	Date of sequence deposition in NCBI	Place of isolation
1	MK315208.1	India	2-APR-2018	23-JUL-2019	Mizoram, India
2	MK315210.1	India	19-APR-2018	23-JUL-2019	Mizoram, India
3	EU807840.1	China	26-JUL-2016	26-JUL-2016	Harbin, China
4	KT988004.1	USA	2006	15-DEC-2015	Ames, USA
5	KX462792.1	USA	23-APR-2012	22-JUL-2017	Greenmead, USA
6	KU318406.1	USA	APR-2015	11-JAN-2017	Greenmead, USA
7	KC469618.1	USA	1995	16-SEP-2013	Brookings, USA
8	AH015834.2	China	2006	05-APR-2016	Beijing, China
9	DQ056373	Thailand	2005	31-MAY-2005	Bangkok, Thailand
10	EU864231	China	06-SEP-2007	26-JUL-2016	Guangdong, China
11	AY032626	China	30-MAY-2001	22-JUL-2016	Harbin, China
12	AB288356	Japan	1992	25-JUN-2008	Tsukuba, Japan
13	EU262603	China	2007	26-JUL-2016	Hubei, China
14	AY366525	USA	19-MAR-2004	26-JUL-2016	Minnesota, USA
15	EU109503	China	12-SEP-2007	12-SEP-2007	Beijing, China
16	AY262352	China	2002	10-NOV-2004	Beijing, China
17	AY457635	China	23-NOV-2003	26-JUL-2016	Hubei, China
18	EF532816	USA	31-MAR-2008	30-MAY-2014	Minnesota, USA
19	DQ473474	Korea	25-SEP-2006	25-SEP-2006	South Korea
20	AF176348	Canada	03-SEP-2002	24-JUL-2016	Ontario, Canada
21	AF184212	Singapore	28-SEP-2000	26-JUL-2016	Singapore
22	EU864233	China	11-Nov-2006	26-JUL-2016	Guangdong, China
23	EU860248	China	OCT-2006	25-APR-2012	Jilin, China
24	EU106888	China	09-SEP-2007	09-SEP-2007	Beijing, China
25	PP227416	Nepal	26-DEC-2022	03-FEB-2024	Udaypur, Nepal

The sequences are arranged according to the gene accession number, countries, year of isolation, year of sequence deposition and place of isolation.

## 3 Results

Out of 180 serum samples collected from different districts, 37 were found positive for PRRSV antibodies resulting in an overall seroprevalence of 20.5% (22). However ORF5, ORF7 and Nsp2 virus sequence was generated from one PCR positive serum sample. Of 35 ORF5 sequences, it was observed that most of the sequences were similar except the sequences isolated from China (HM755885.1), South Korea (KT257724.1 and KT988136.1), Australia (AY615793.1 and AY615790.1) and Spain (KF666936.1). These sequences were longer at 5′ end by 15 nucleotides. Similarly, of 30 ORF7 sequences, it was also observed that Spain (MK024325.1, OM893855.1, GU067771.1, and DQ057992.1), China (HM755885.1), Russia (KX668221.1), and Vietnam (KM659203.1) were different as compared to the other sequences. The phylogenetic analysis of Nsp2 revealed that the strain isolated from Nepal is clustered into Indian isolate and Chinese isolates. All these sequences were elongated by 12 nucleotides except Vietnam which was elongated by 48 nucleotides at the 3′ end. This indicated that the sequences of PRRSV throughout the world are variable.

Blast analysis of ORF5 sequences of Nepal with that of reference strain showed that the nucleotide sequence identities ranged from 98.34% to 84.31% and the amino acid similarities ranged from 99% to 58.79%. It shares 98.34% to 77% nucleotide identity with Indian isolates, isolate no. MT347587.1, MK764031.1, MT274643.1, and KT844658.1. The sequence identity of ORF5 shares 96.52% to 96.35% with the Chinese isolates. Similarly, the nucleotide sequence identities of ORF7 ranged from 100% to 90.05% and the amino acid similarities ranged from 100% to 60.91%. ORF7 sequence identity with the Indian isolates ranged from 100% to 97.58% and with the Chinese isolates ranged from 97.58% to 97.31%. Blast analysis of Nsp2 sequence revealed that the Nsp2 nucleotide identities ranged from 92.85% to 80.03% and amino acid similarities ranged from 91.59% to 41%.

### 3.1 Phylogenetic tree

From the phylogenetic tree constructed from 35 sequences of ORF5 (Figure 2), 30 sequences of ORF7 (Figure 3), and 25 sequences of Nsp2 (Figure 4) it was observed that the Nepalese strain of PRRSV belongs to the PRRSV-2 species. It can be observed that the strain identified in Nepal is closely related with the strains isolated from India in 2020 and 2018, and belongs to lineage 8 as defined by Shi et al. (15).

## 4 Discussion

The first outbreak of PRRSV in Nepal occurred in a national pig farm of Khumaltar in 2013 showing the signs of abortion and stillbirth in sows (20). Since then, the disease is spreading widely all over the country causing a huge impact in the national economy. Field observation in the farm showed the poor biosecurity measures, lack of PRRSV knowledge and awareness among farmers. Furthermore, the practice of keeping both seropositive pigs and the recovered pigs by farmers has led to the persistence of infection in the animal population increasing the risk of spread of disease (22). Despite the severe economic loss caused by this disease, adequate studies on molecular epidemiological has not been conducted. This study has attempted to characterize the PRRSV based on ORF5, ORF7, and Nsp2 from the samples collected during PRRSV outbreak in different districts of the country in 2021.

ORF5/ORF7 sequence analysis have been widely used in PRRS genotyping showing ORF dependent clustering in phylogenetic trees with some possible recombination (24, 25). ORF5 having a mutation rate of approximately 0.5%–1% per year, consists of approximately 600 nucleotides which shows great genetic diversity (24, 26). Even the isolates sharing identical genotypes can exhibit significant variations in their genetic sequences, particularly within the Nsp2 gene and ORF5 region (27). The phylogenetic analysis of Nsp2 revealed that the strain isolated from Nepal is clustered into India and China. Although whole genome sequencing of PRRSV may provide more complete information, this study could not generate complete PRRSV sequence as there was not sufficient viable RNA in the sample. However, sequencing of ORF5, ORF7, and Nsp2 has provided sufficient information to determine phylogenetic inferences. The sequences from a PRRSV strain from Nepal revealed a high degree of similarity with the strains isolated from India, China, and Vietnam, with the closest genetic relatedness to the Indian isolates from 2020 and 2018. From the phylogenetic tree, it can be inferred that the strain identified in Nepal was closely related to the strains isolated from India whereas studies have shown that the Indian isolates resembles to HP-PRRSV of China (21). Likewise, PRRSV isolate of Nepal shares 97.01% nucleotide identity with the Indian strain isolated during the outbreak in Mizoram state, India. Though the analysis of additional samples is required to provide the complete information about all the strains circulating in Nepal and their source, this study has demonstrated for the first time in Nepal the active circulation of a PRRSV-2, lineage 8 strain in domestic pigs based on ORF5, ORF7 and Nsp2 sequence analysis.

PRRSVs are rapidly evolving RNA viruses which have highly affected the pig industry worldwide. Emergence of new highly pathogenic PRRSV strains leads to widespread outbreak of PRRS. In Nepal, the disease could be spreading mainly through occasional movements of pigs from one place to another, via contaminated vehicles, and via semen from infected boars. The porous international border with India also contributes to transmission of the disease where the animals and animal products are transported without any barrier. Studies have indicated that wild boars, being a reservoir of PRRSV, may act as a source of infection for domestic pigs (28, 29). However, the status of PRRSV infection in wild boars of Nepal is yet to be revealed.

Control of PRRSV is not only important but also very challenging due to the high genetic variability of the virus (30). Till now, neither national control measures nor compensation to the farmers have been implemented in Nepal despite the disease-causing major constraints to the pig production and productivity. All-in all-out measures, conducting PRRSV diagnostic testing before the introduction of animals or semen into the farm and strict biosecurity measures seems only the possible means to prevent the introduction of the disease.

PRRSV genotyping is one of the major tools to better understand the virus ecology. The present study demonstrates that a PRRSV strain from Nepal belongs to the PRRSV-2 species (previously North American genotype). Since the disease has a high economic impact, monitoring and genotyping of circulating viruses is an important tool in order to improve diagnostics and increase vaccine efficacy.

## 5 Conclusion

Here a PRRSV-2 phylogenetic analysis was conducted and described based on ORF5, ORF7 and Nsp2 sequence analysis. A field strain from this study belonged to lineage 8. Further studies are very necessary to elucidate its geographic distribution, dynamic of genotypes and potential vaccine implementation.

## Data availability statement

The datasets presented in this study can be found in online repositories. The names of the repository/repositories and accession number(s) can be found in the article/supplementary material.

## Ethics statement

This study was approved by Nepal Agricultural Research Council. Written informed consent was obtained from the owners for the participation of their animals in this study.

## Author contributions

MP: Conceptualization, Investigation, Writing – original draft. MA: Data curation, Writing – review & editing. YL: Writing – review & editing. ZZ: Writing – review & editing. MPA: Project administration, Writing – review & editing. SC: Methodology, Writing – review & editing. J-PF: Data curation, Methodology, Writing – review & editing.
